# Experimental study on the freezing process of water droplets for ice air jet technology

**DOI:** 10.1038/s41598-024-53730-9

**Published:** 2024-02-08

**Authors:** Hu Jingru, Li Jingbin, Huang Zhongwei, Cheng Kang, Xia Haojun

**Affiliations:** State Key Laboratory of Petroleum Resources and Prospecting, China University of Petroleum, Beijing, 102249 China

**Keywords:** Ice air jet, Abrasive jet, Visualization, Cleaning, Processing, Environmental sciences, Engineering

## Abstract

Ice air jet technology is one non-destructive, residue-free and environmentally friendly machining process. It is an efficient method to form ice particles by atomizing water droplets in ultra-low temperature environment. The freezing characteristics of water droplets in cryogenic gas and liquid nitrogen environment are visually analyzed, and the effects of droplet volume, ambient temperature are also studied. The results show that when water droplets freeze in a cryogenic gas environment, four distinct stages are observed, namely pre-cooling stage, recalescence stage, solidification stage, and deep cooling stage. However, when water droplets freeze in liquid nitrogen, the recalescence stage cannot be observed. For a 5 µl water droplet, it takes 68 s for water droplets to freeze into ice particles at − 20.36 °C, while it takes only 1.7 s in liquid nitrogen. During the freezing process, the water droplets form an ice shell outside and freeze inwardly. Ice particles may break up due to differences in solubility and density. With the increase of volume the time spent on pre-cooling stage and freezing stage both increases. For the large latent heat of water phase transformation, the solidification stage time is greatly affected by the volume of water droplets. When the ambient temperature drops from − 10.67 °C to − 24.68 °C, the freezing time of 5 µl water droplets decreases by 45.5%, indicating that the ambient temperature has a great influence on the freezing time. The results of the study can significantly contribute to the development of ice air jet technology.

## Introduction

Abrasive water jet (AWJ) cleaning technology aims to improve the processing performance or polishing efficiency of existing water jet processing technology by adding small abrasive particles to the stream of high-speed water jet^1,2^. AWJ technology is successful utilized in cutting, cleaning, machining, and surface preparation operations. However, a considerable amount of secondary particle waste and contamination impingement by abrasive materials have been an important issue^3–6^. AWJ is not suitable for applications such as processed meat, medical surgery, cleaning sensitive surfaces and so on. Dry ice blasting is an efficient and environmentally friendly method for cleaning contaminated surfaces^7,8^. But dry ice particles are generally rod-like, and the preparation and preservation costs are high. Therefore, the development of an environmentally friendly and efficient surface treatment is becoming more and more intense.

Ice Air Jet (IAJ) technology is a process in which particles of solid ice are propelled at high velocity to impact and clean a surface. IAJ is a non-destructive, residue-free and environmentally friendly processing technology^9^. By substituting ice particles for the abrasive particles, it is possible to combine process efficiency with environmental benefits. It ensures good eco-friendly material processing and lower costs, allowing for inclusive and long-term multi-industry cleaning and polishing operations. In 1980s, Galecki and Vickers^10^ obtained ice particles by crushing ice cubes, and proved that the effect of air-ice jet was better than that of water jet, and its effect could meet technical requirements of most surface treatments. After that, Geskin et al.^11,12^ demonstrated the feasibility of ice particles as a substitute for mineral abrasives and developed a system for producing ice particles by crushing ice. At same time, Shishkin^13^ designed and demonstrated the corresponding technology for the application of ice powder for material processing. In 2013, Li et al.^14^ made ice particles by making water droplet directly contact with the atomized liquid nitrogen, in which water droplets can quickly condense into cold ice particles. In 2020, Marko et al.^15^ evaluated the erosion capability of ice particles by blasting the aluminium and glass surfaces, which showed that ice particles have the potential to generate similar damage in the workpiece as garnet. In 2022, Li et al.^16^ relized instant preparation of ice particles on a vertical low-temperature wall using the principle of heterogeneous nucleation.

Although the "green" ice jet process is highly desirable, as a new jet technology, there are still many scientific problems and technical difficulties to be solved. The first problem is how to obtain high quality ice particles. Because the ice particles can exist only at subzero temperature and tend to stick together, so it is necessary to develop a practical technology for ice generation^17,18^. The methods of ice particles preparation are broadly divided into two categories: one is the preparation of ice particles by ice crushing; the other is the production of ice particles in real time by atomizing high-pressure water under an ultra-low temperature environment^19,20^. The process of ice preparation by mechanical ice crushing is generally to freeze ice of suitable size first, then crush and then sieve out the ice particles of the required size^21^. This method is simple and easy to implement, but the disadvantage of the broken ice method is not continuous production of ice particles and in the process of crushing and sieving ice particles is easy to melt and adhesion^22^. Ice particles can be obtained continuously by mixing atomized liquid nitrogen and atomized water droplets. The distinctive feature of this method is that the size and uniformity of the atomized water droplets can be controlled by changing the atomization nozzle or by other means, such as ultrasonic atomization^23–25^. In this method, water droplets are immediately frozen into small ice particles by contacting with cryogenic nitrogen. Castillo^26^ used the infrared (IR) thermography to quantify the fraction of latent heat released to the substrate and the ambient air. Similarly, Bodaghkhani^27^ investigated the total freezing time of droplets on surfaces with various wettabilities with horizontal and inclined orientations by experimental and simulations.

The efficient preparation of high quality ice particles is the key of IAJ technology. Therefore, we carried out visualization experiments to reveal the freezing process and mechanism of a single water droplet. The effects of droplet volume, ambient temperature and heat transfer medium on water droplet freezing were also studied. Research results lay a solid foundation for future research of IAJ.

## The basic theory of freezing water droplets

When water droplets are exposed to low temperatures, due to the effect of temperature difference, water droplets will continue to release heat until they condense into ice particles and cool down to the ambient temperature. In this physical process, there are four stages: pre-cooling stage, recalescence stage, solidification stage, and deep cooling stage^28^.

**Figure 1:**
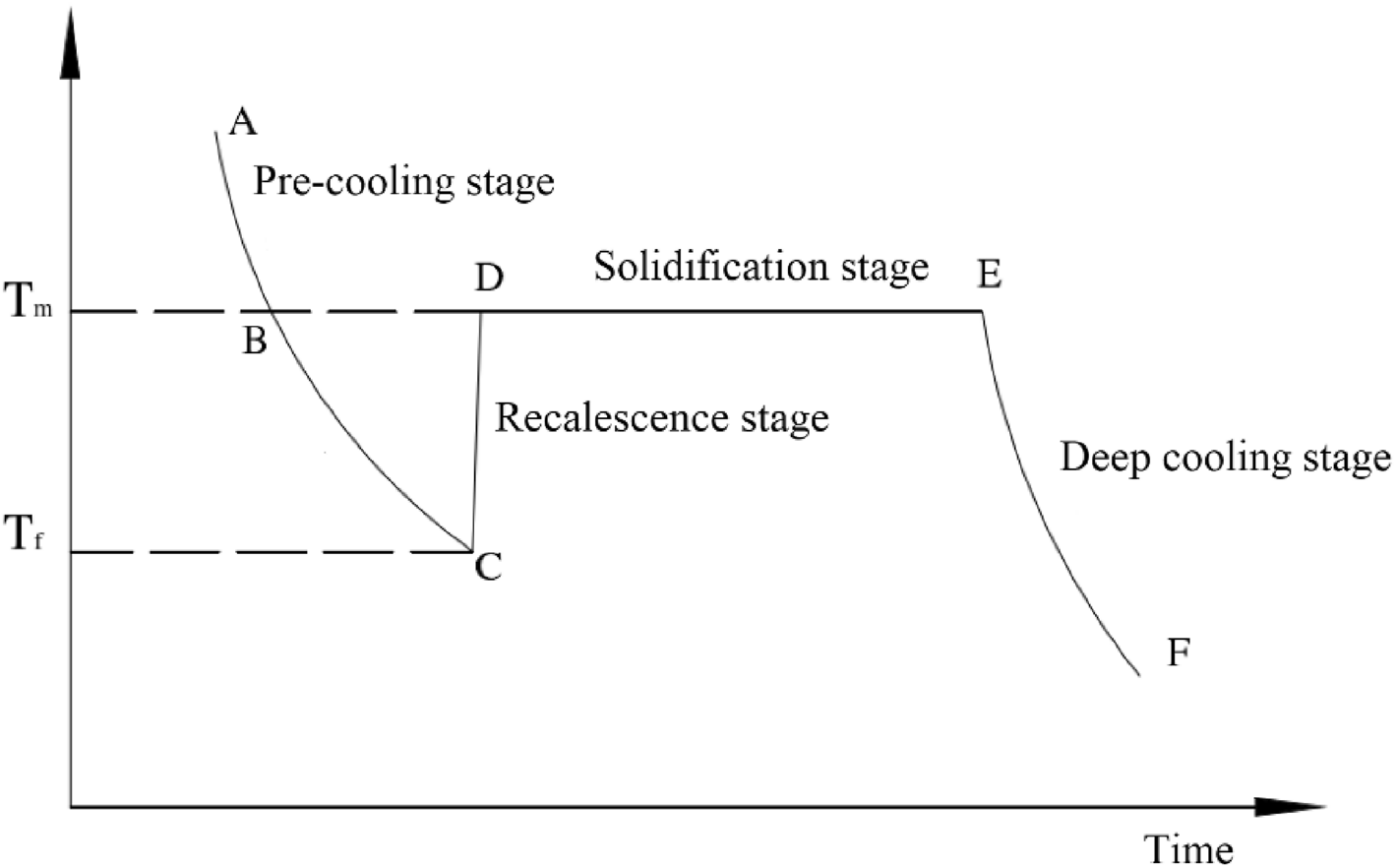
Temperature curve of water droplets during freezing into ice particles.

As shown in Fig. 1, in the pre-cooling stage, the water droplets are always in the liquid state and the temperature gradually decreases. It should be noted that the water droplets will enter a supercooled state, that is, under atmospheric pressure conditions, the temperature of the water droplets is below zero, but it will remain liquid. The supercooled state is a kind of metastable state, and the external slight disturbance can easily lead to the formation of crystal nuclei in water droplets and the release of the supercooled state. The phase variation of water droplets occurs at point C, so the temperature at point C is called the nucleation temperature (*T*_*f*_). In this process, heat transfer is mainly carried out through the surface heat conduction, water droplet evaporation and heat radiation.

The sudden return of the temperature of the water droplets from the nucleation temperature to the melting temperature of ice is called the recalescence stage. During this process, due to the continuous effect of low-temperature, crystal nuclei are formed in the supercooled water droplets, and a part of the liquid changes phase instantly to form ice. At the same time, the latent heat released by the phase variation makes the temperature of the water droplets quickly return to the freezing temperature of the water or the melting temperature of ice (*T*_*m*_, 0 °C, atmospheric pressure), and the whole process is very short.

During the solidification stage, the liquid part of the water droplets gradually solidifies into ice and continuously releases latent heat, keeping the core temperature of the water droplets at 0 °C, until whole water droplets are completely frozen and solidified into ice.

When the water droplets are completely frozen, under the action of low temperature environment, the temperature of the ice particles gradually decreases to the ambient temperature, this process is called the deep cooling stage.

## Experiment setup

### Equipment

**Figure 2:**
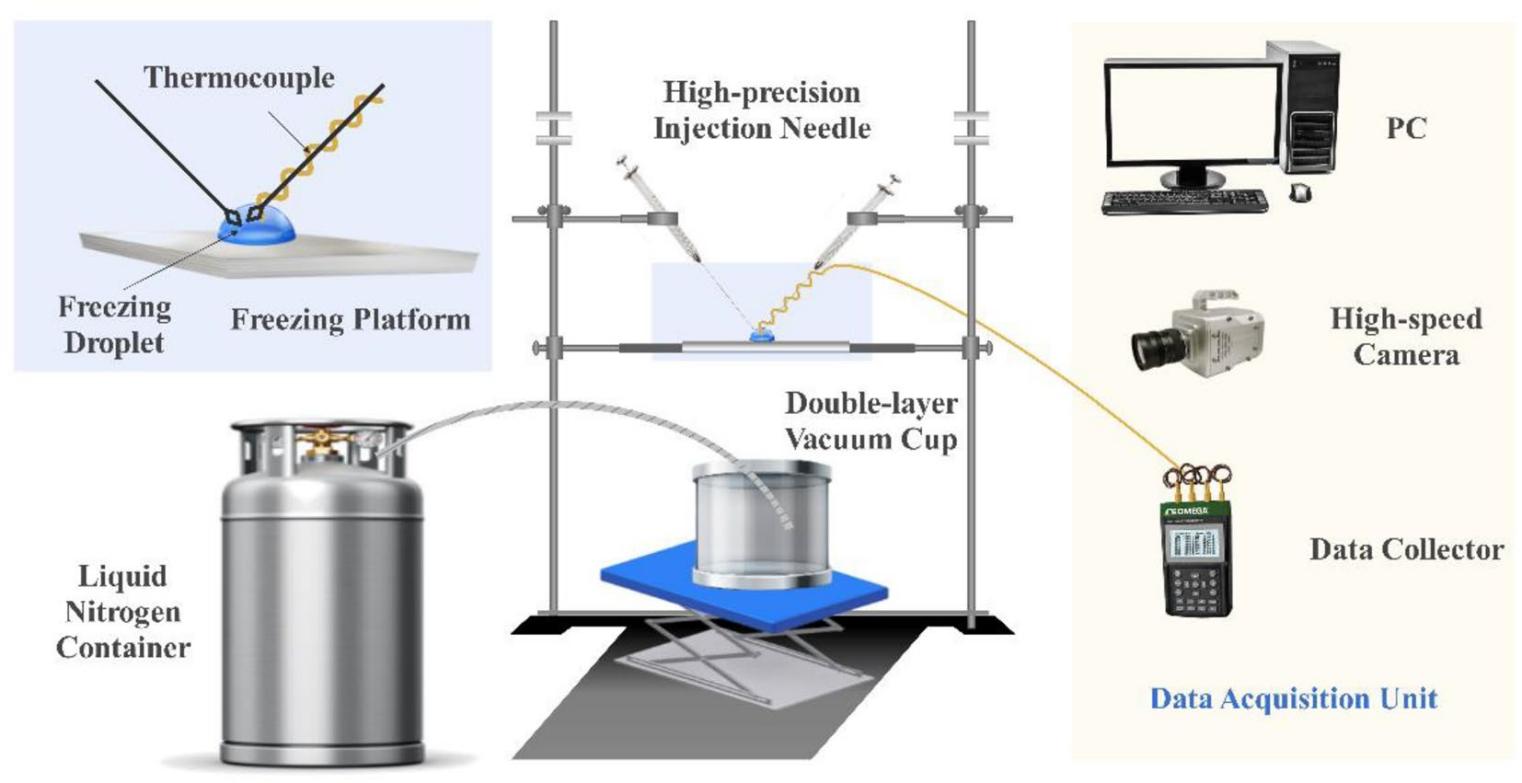
Schematic diagram of visual experimental system.

In order to study the solidification process of a single droplet, a visual experimental platform was developed. The platform includes the freezing platform, data collector, thermocouple, high-speed camera, high-precision injection needle, transparent double-layer vacuum cup, and liquid nitrogen container. The schematic diagram is shown in Fig. 2. The high-precision injection needle with a minimum scale of 0.1 µl designed by Hamilton is used to produce water droplets with different volumes. The water droplet is placed at the freezing platform and raised into right position. Here, a 0.125 mm omega T-type thermocouple is used to measure the temperature change of water droplets, and its accuracy is 1.0 °C or 1.5% at low temperatures (below 0 °C). The ends of the thermocouple are welded together and inserted into the center of the water droplet. The OMEGA data collector was used to collect the temperature variation rule with time, and its sampling frequency was 500HZ. The I-speed 726 (ix camera) high-speed camera was used to observe the morphology changes of water droplets. When the resolution is 1080P, the frame rate is 12,742fps, and the highest frequency is 2.45 million fps.

### Methodology

#### Temperature variation during the water droplet freezing process

Experiments were conducted to study the temperature variation during the water droplet freezing process. First, a certain amount of liquid nitrogen is poured into a double-layer vacuum cup. Due to the rapid evaporation of liquid nitrogen, a low temperature environment is formed at the upper part of double-layer vacuum cup. By moving the position of the freezing platform, adjusting its distance from the liquid nitrogen surface, finding the location of the set temperature, and fixing it. Then, the double-layer vacuum cup is removed and coverd, and a specific volume of water droplets is placed on the freezing platform with the high-precision feeding needle. And put the thermocouple welding at the center of the droplet. At last, place the vacuum double layer cup at the set position. At low temperature environment, the water droplet begins to solidify, and its internal temperature is recorded by the thermocouple.

#### Visualization experiments of the freezing process

The morphologies of water droplets frozen in low temperature enviroment were observed. The experimental procedure is the same as above, the only difference is that the temperature is no longer measured, but only the morphological change is observed by high-speed photography. In addition, by dropping water droplets directly into liquid nitrogen, we observed the solidification process of water droplets in liquid nitrogen.

#### Uncertainty analysis

Since the double-layer vacuum cup is open, and the low temperature environment is formed through the evaporation of liquid nitrogen, there will be some difference between the actual temperature and the set temperature. However, it can be verified from the final temperature of ice particles, and the temperature difference is within ± 1 °C, which has little influence on the experiment. It is precisely because of the low temperature environment created by liquid nitrogen evaporation, the influence of environmental temperature, humidity and other parameters was not considered in the experiment. In addition, under the action of gravity and surface tension, the water droplets resting on the frozen platform are not perfectly round, and the placement of thermocouples is difficult to ensure that the center of the water droplets. The resulting error can be reduced by repeated experiments.

### Experiment scheme

In order to fully understand the temperature and morphology changes of water droplet during freezing, the volume of water droplets is selected as 4, 5 and 6 µl. In order to study the freezing process under different temperature conditions, the temperatures were set as − 15 °C, − 20 °C and − 25 °C respectively. In addition, the difference of water droplets in low temperature gas and liquid nitrogen (− 196 °C) was studied too. Details are shown in Table 1.

**Table 1:** Experiment project.

Contact method	Volume (µl)	Environment temperature (°C)
Gas–liquid	4/5/6	− 15/− 20/− 25
Liquid–liquid	4/5/6	− 196 °C

## Results and discussions

### Freezing characteristics of water droplets in low temperature gas environment

#### Central temperature variation during water droplets freezing

**Figure 3:**
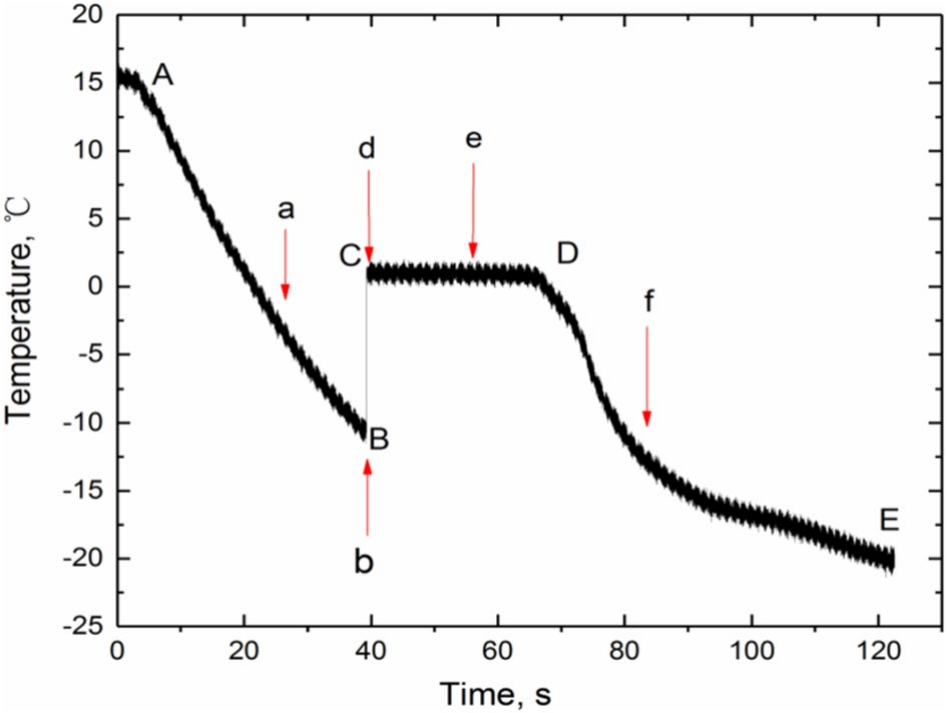
Central temperature variation of water droplets during freezing process.

Figure 3 shows the central temperature variation of a 5 µl water droplet at − 20.36 °C. Four stages, pre-cooling stage (shown in AB), recalescence stage (shown in BC), solidification stage (shown in CD), and deep cooling stage (shown in DE), can be observed clearly. In the pre-cooling stage, once the water droplets enter the low temperature environment, they will be rapidly cooled from orginal temperature to the supercooled temperature (about − 10 °C). Water droplet keeps the liquid state in this stage and the process holds around 35 s. At this stage, there is no phase transformation, and the water droplet exchange its own heat through heat conduction, convection and radiation. Then crystal nucleation occurs and the freezing process comes into recalescence stage which is usually very short and only lasts for about 10 ms. At this stage, water droplets undergo phase transformation, releasing latent heat and restoring the temperature of water droplets to the melting temperature (usually 0 °C for ice). After that, the process comes into the solidification stage. The temperature of the droplet keeps around 0 °C and this stage ends after the 30 s until the droplet completely frozen. At this point, the droplets have completely frozen into ice particles in about 68 s. Finally, at the deep cooling stage, the ice grain is reduced to ambient temperature.

#### Morphology change during water droplets freezing

**Figure 4:**
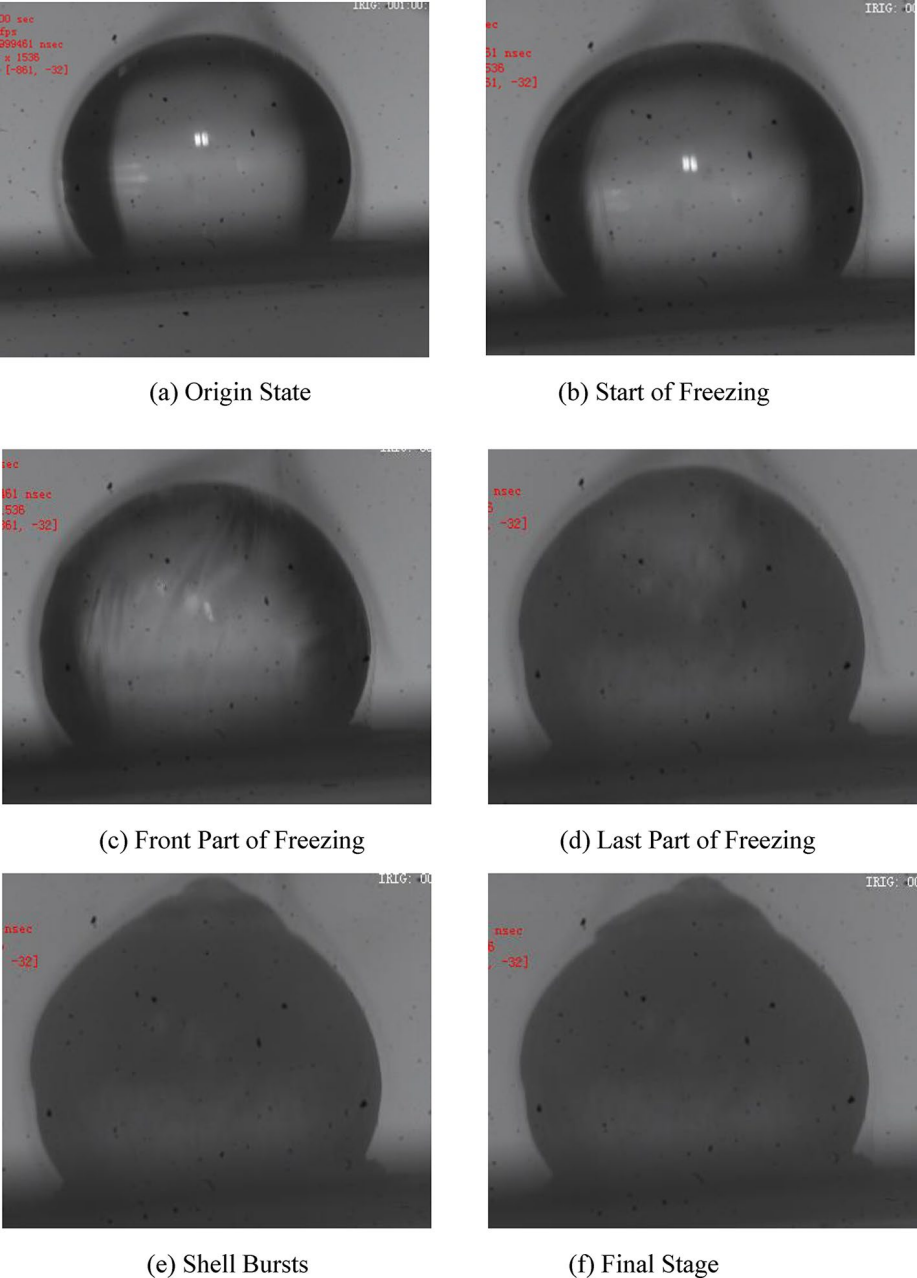
Video frames of the water droplet during freezing.

The morphological changes of water droplets during freezing were recorded by high-speed photography (shown in Fig. 4). Transparent water droplet with a liquid state at the beginning can be observed clearly in Fig. 4a. In Fig. 4b, the droplet outside begins to obscure and form a shell. The shell gradually covers the water droplet in Fig. 4c. In Fig. 4d, the shell completely formed and became opaque. The top of the droplet gradually rises and deforms. Figure 4e,f) show that a corner can be clearly observed and even break the top of the droplet. This is due to the solubility difference between ice and water, some air escape from the liquid and gradually accumulate and break the ice shell. Besides, the breaking of ice particles can also be caused by volume change since the density of water is greater than ice. In other word, during the freezing process in low temperature gas environment, ice shells will form on the surface at first, and then gradually freeze inward, ice particles may break at the top. In addition, it can also be concluded that the volume of water droplets should not be too large, otherwise ice particles will break. On the other hand, the volume of water droplets should not be too small, otherwise the impact force will be insufficient. Therefore, the volume of water droplets has an optimal value.

### Freezing characteristics of water droplets in liquid nitrogen

#### Central temperature variation during water droplets freezing

**Figure 5:**
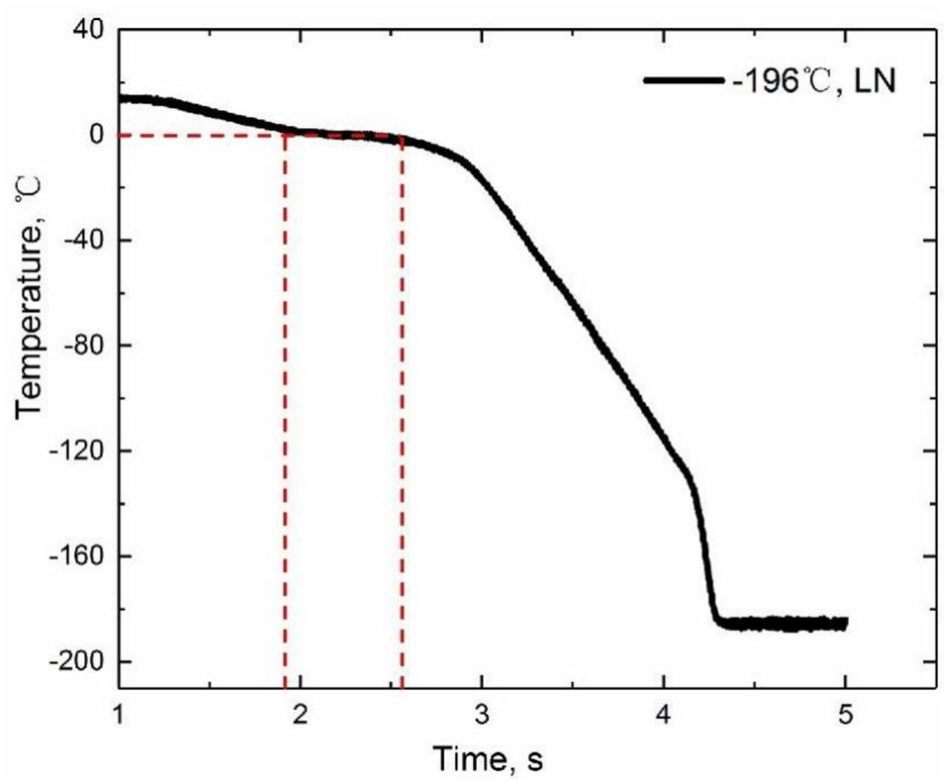
Central temperature variation of water droplets during freezing process.

Water droplets were immersed into liquid nitrogen directly to reveal the heat transfer mechanism in liquid nitrogen. Figure 5 shows that the temperature variation of a 5 µl water droplet in liquid nitrogen. Only three stages, the pre-cooling stage, solidification stage, and cooling stage, can be observed clearly. It is also find that the freezing stage can be completed within a very short period (about 1.7 s). The reason that only three stages are observed can be classified as follows: First, the temperature difference between the water droplet and liquid nitrogen is too large which exceeds 200 °C. In addition, the heat transfer medium changed. The previous experiment was conducted in air, which had a low thermal conductivity, while this experiment was conducted in liquid nitrogen, which had a high thermal conductivity, so the heat transfer was faster. It is very hard to accurately calculate the freezing time of the droplet freeze in liquid nitrogen, but Li et al.^29^ estimate the time costs for phase change of a droplet with 0.15 mm diameter will last 6 ms. According to Duan's method, a droplet with 5 µl whose radius is 1.5 mm and freezing in liquid nitrogen will spend 0.32 s in finishing phase change.

#### Morphology change during water droplets freezing

**Figure 6:**
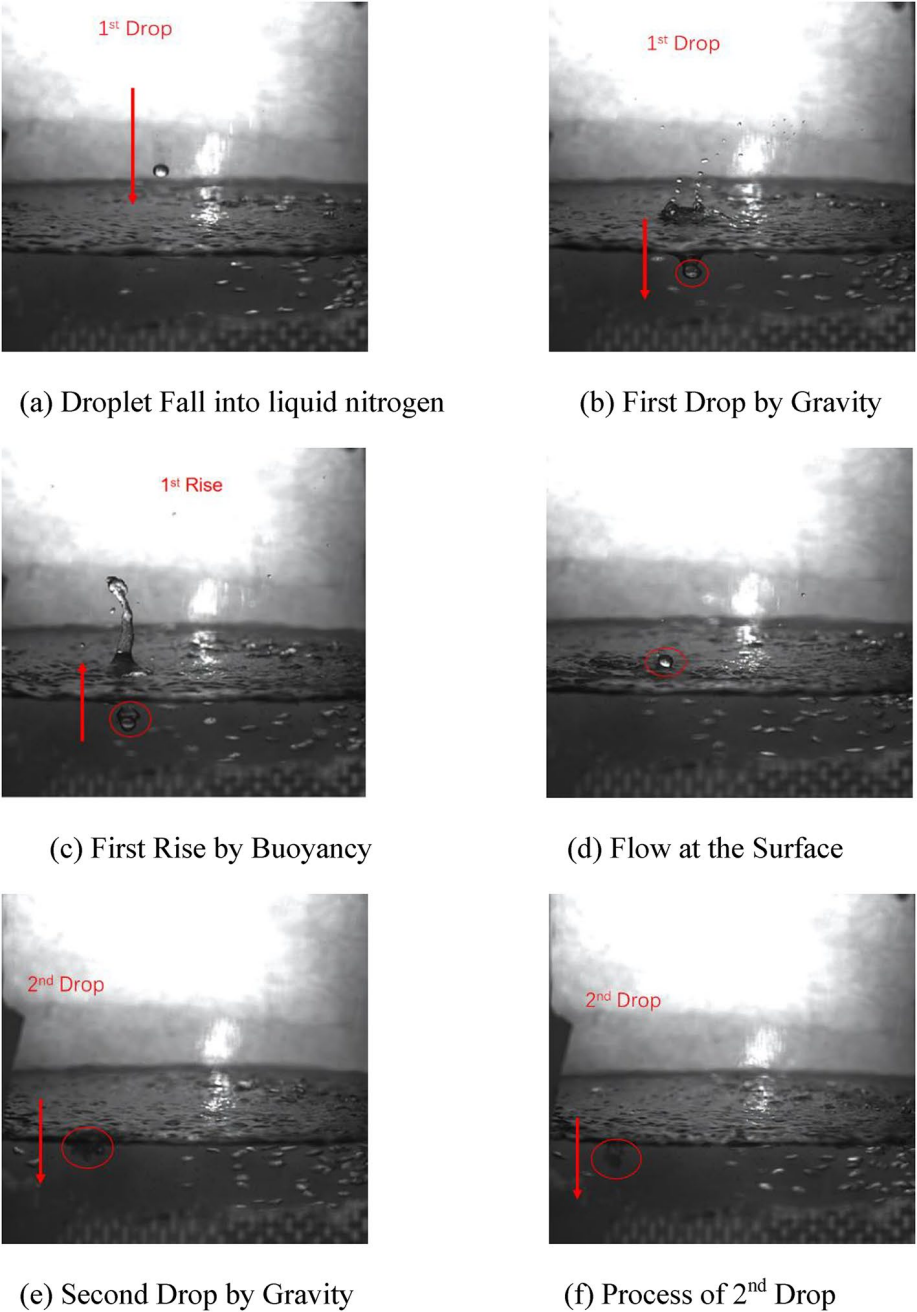
Physical motion of water droplet fall into liquid nitrogen.

By dropping water droplets directly into liquid nitrogen, the freezing characteristics of water droplets in liquid nitrogen were studied. It is found that although the density of water is greater than that of liquid nitrogen, after water droplets are added to liquid nitrogen, they will move irregularly on the surface of liquid nitrogen. As shown in Fig. 6, when a water droplet falls into liquid nitrogen for the first time, it is immersed in the liquid nitrogen by inertia (Fig. 6a–c), but soon bounces back to the surface and begins to behave irregularly until it freezes completely and changes from transparent to opaque (Fig. 6d–e), and finally sinks back into the liquid nitrogen again (Fig. 6f). This process lasts for more than ten seconds. The Leidenfrost Phenomenon could explain the random motion of water droplets in liquid nitrogen^30,31^. The huge temperature difference between the water droplet and liquid nitrogen can speed up the liquid nitrogen vaporization and form a thin gas film. The vaporized nitrogen wraps around and drives the water droplets in random motion^32^.

### The influence of water droplet volume on freezing process

**Figure 7:**
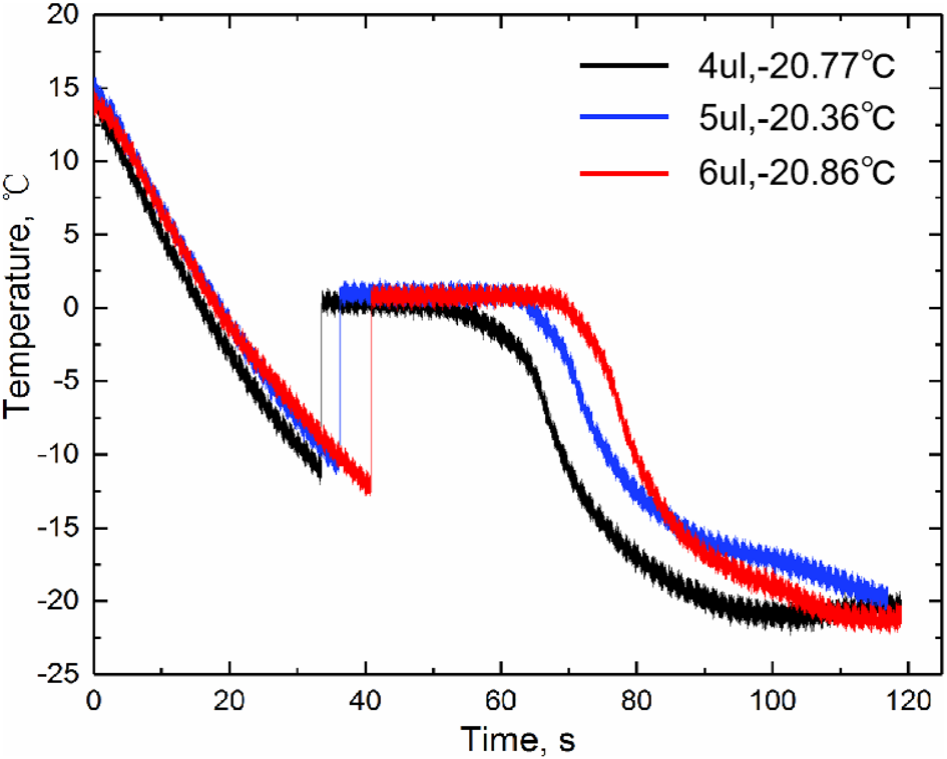
Central temperature variation of water droplets with different vloume during freezing process.

Freezing experiments of 4 µl, 5 µl and 6 µl water droplets were carried out respectively, with corresponding droplet diameters of 1.44 mm, 1.55 mm and 1.65 mm. As shown in Fig. 7, four stages of droplet freezing can be obviously observed. The time spent in each stage is different, as shown in Table 2. Former experiments have shown that water dropet can be frozen completely at the end of the soldification stage and the recalescence stage only last less than 10 ms. Therefore, only the pre-cooling stage and freezing stage are analyzed here. With the increase of volume the time spent on the two stages also increases. The total freezing time is about 60 to 70 s, while the deep cooling time is nearly 60 s. Because the latent heat of water phase variation is large, so the solidification stage time is greatly affected by the volume of water droplets. Combined with the visual experiment results, there is an optimal water droplet volume, which needs further study.

**Table 2:** Time for droplet with different volume freezing.

Material	Volume (µl)	Environment temperature, (°C)	Time for Pre-cooling (s)	Time for freezing (s)
Water	4.00	− 20.77	33.10	14.16
	5.00	− 20.36	38.20	24.19
	6.00	− 20.86	41.04	25.17

### The influence of environmental temperature on the freezing process

**Figure 8:**
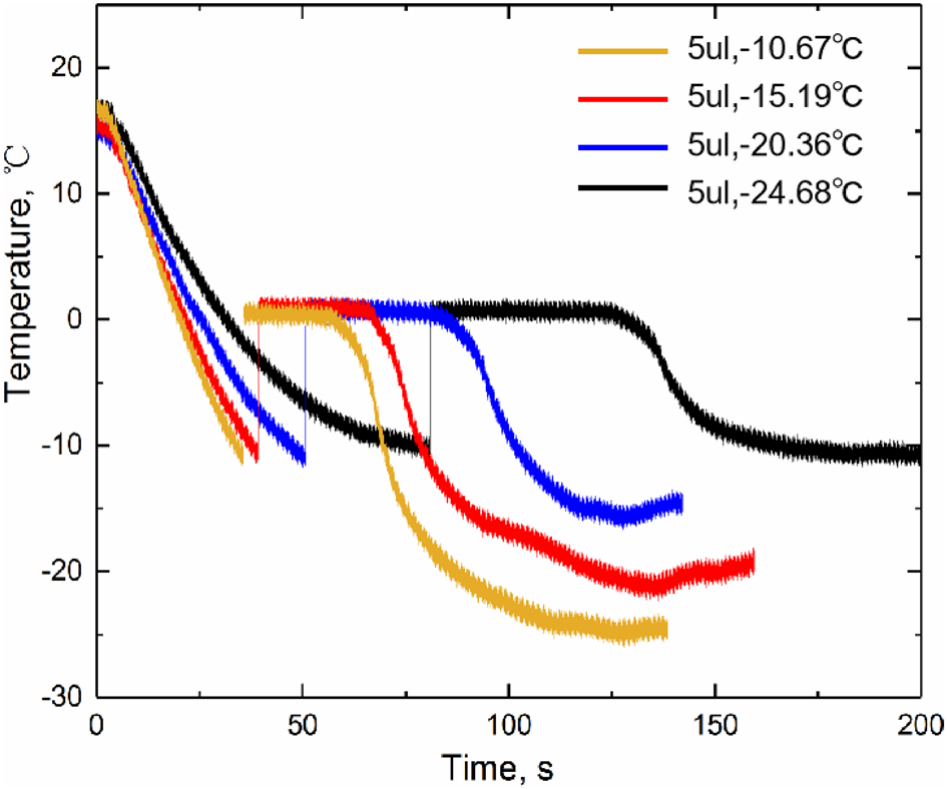
Central temperature variation of water droplet during freezing process in different temperature.

The freezing experiments of a 5 µl water droplet in four different temperature gas environment (− 10 °C, − 15 °C, − 20 °C, − 25 °C) were carried out. The temperature change curve during freezing is shown in Fig. 8. With the decrease of ambient temperature, the pre-cooling time and freezing time both decrease, and the final temperature also decreases. The pre-cooling stage costs 79.63 s and the freezing stage costs 43.46 s for a 5 µl water droplet freezing in − 10.67 °C and the data is 34.12 s and 21.92 s respectively − 24.68 °C. When the ambient temperature is reduced by 15 °C, the freezing time is reduced to 45.5% of the original, indicating that the ambient temperature has a great influence on the freezing time. But the cost of creating a low-temperature environment is high. Liu et al.^33^ found that abrasive hardness is the main factor affecting the erosion effect. According to the study of Taoshiaki^34^, at − 50 ~ − 60° C, the Mohs hardness of ice particles is about 4, which can meet the needs of engineering applications. At the same time, according to Hobbs' research^35^, when the ice grain temperature is lower than − 27 °C, the adhesion between ice grains is zero, that is, ice grains will not bond during transport. Therefore, it is recommended that the ambient temperature for preparing ice particles is − 50 ~ − 60 °C.

## Results

Ice jet technology (IAJ) is one non-destructive, residue-free and environmentally friendly machining process technology. Through the freezing experiments under different conditions, the solidification mechanism, morphology changes and the influence of key parameters are studied, which can strongly promote the development of ice jet. Results are concluded as follows.

1. When a water droplet freezes in cryogenic gas environment, pre-cooling stage, recalescence stage, solidification stage, and deep cooling stage were observed. At − 20.36 °C, the freezing time is about 68 s. However, when water droplet freeze in liquid nitrogen, the recalescence stage cannot be observed. The freezing stage can be completed within a very short period (about 1.7 s).
2. During the freezing process, the water droplet will form an ice shell outside and freeze inwardly. And ice particles may break up due to difference in solubility and density between water and ice. When water droplets freeze in liquid nitrogen, they will first move irregularly on the surface of the liquid nitrogen due to the Leidenfrost Phenomenon, and then sink to the bottom of the liquid nitrogen after completely freezing.
3. With the increase of volume the time spent on pre-cooling stage and freezing stage both increases. For 4–6 µl water droplets in − 20 °C environment, the total freezing time is about 60 to 70 s, while the deep cooling time is nearly 60 s. For the large latent heat of water phase transformation, the solidification stage time is greatly affected by the volume of water droplets. Combined with the visual experiment results, there is an optimal water droplet volume, which needs further study.
4. With the decrease of ambient temperature, the pre-cooling time and freezing time both decrease, and the final temperature also decreases. When the ambient temperature drops from − 10.67 °C to − 24.68 °C, the freezing time of 5 µl water droplets decreases by 45.5%, indicating that the ambient temperature has a great influence on the freezing time. Considering the factors such as economy, hardness and cohesiveness of ice grains, it is recommended that the ambient temperature for preparing ice particles is − 50 ~ − 60 °C.

## Data Availability

The datasets used and/or analysed during the current study available from the corresponding author on reasonable request.
